# Why Children Die

**Published:** 1859-06

**Authors:** 


### WHY CHILDREN DIE.

"I have seen persons who gather for the parlor their choicest flowers, just as they begin to open into full bloom and fragrance, lest some passer-by should tear them from the bush and destroy them. Does not God sometimes gather into heaven young and innocent children for the same reason—lest some rude hand may despoil them of their beauty."

Some weak brother has been trying his hand to see what a beautifully sounding sentence he could make out of a whopper. The reason why children die is because they are not taken care of. From day of birth they are stuffed with food, choked with physic, sloshed with water, suffocated in hot rooms, steamed in bed clothes. So much for in doors. When permitted to breathe a breath of pure air once a week in summer, and once or twice during the colder months, only the nose is permitted to peer into daylight. A little later they are sent out with no clothing at all, as to the parts of the body which most need protection. Bare legs, bare arms, bare necks; girted middles, with an inverted umbrella to collect the air, and chill the other parts of the body. A stout strong man goes out on a cold day with gloves and overcoats, woolen stockings and thick double soled boots with cork between and rubbers over. The same day, a child of three years old, an infant in flesh and blood, and bone and constitution, goes out with soles as thin as paper, cotton socks, legs uncovered to the knees, arms naked, necks bare; an exposure which would disable the nurse, kill the mother outright in a fortnight, and make the father an invalid for weeks. And why? To harden them to a mode of dress which they never are expected to practice. To accustom them to exposure, which a dozen years later would be considered downright foolery. To rear children thus for the slaughter pen, and then lay it on the Lord, is too bad. We don't think that the Almighty has any hand in it. And to draw comfort from the presumption that he has any agency in the death of a child, in the manner of the quoted article, is a presumption and a profanation.

